# Migraine and Pregnancy

**DOI:** 10.3390/life14101224

**Published:** 2024-09-25

**Authors:** Katherine Phillips, Prut Koonalintip, Benjamin R. Wakerley

**Affiliations:** 1 Institute of Applied Health Research, University of Birmingham, Birmingham B15 2TT, UK; 2 Division of Neurology, Department of Internal Medicine, Prince of Songkla University, Hatyai 90110, Songkhla, Thailand; 3 Department of Neurology, University Hospital Birmingham, Birmingham B15 2GW, UK; 4 Metabolic Neurology, Institute of Metabolism and Systems Research, College of Medical and Dental Sciences, The Medical School, University of Birmingham, Birmingham B15 2TT, UK

**Keywords:** pregnancy, migraine, headache, miscarriage, pre-eclampsia

## Abstract

Migraine affects almost one in five women of reproductive age. Therefore, understanding its impact on pregnancy outcomes and how to manage migraine safely in pregnancy are of particular importance. This review will summarise the clinical course of migraine during pregnancy, the management of women presenting with headaches during pregnancy, the management of migraine during pregnancy and summarise what is known about how migraine and migraine medications impact pregnancy outcomes.

## 1. Introduction

Migraine affects 1 in 7 people worldwide and is associated with a significant reduction in quality of life [1]. Women are between two and three times more likely to develop migraine and almost a third will experience an attack before the age of 45 years old [1].

Migraine attacks are characterised by moderate to severe headaches and associated with symptoms such as photophobia (sensitivity to light), phonophobia (sensitivity to sound), nausea or vomiting [2]. Up to a third of patients also experience aura before the onset of headache, which is typically visual or sensory and less commonly associated with weakness. Patients who regularly have more than 15 days of headache (of which at least eight of these must have migraine symptoms) are said to have chronic migraine, while those with less have episodic migraine [2].

Migraine is not a benign disorder and, especially when associated with aura, carries increased cardio- and cerebrovascular risk [3,4]. Whilst the vast majority of women with pre-existing migraine report a remission in symptoms during pregnancy, there is a significant body of evidence to suggest that migraine has a negative effects on pregnancy outcomes including the risk of developing pre-eclampsia and miscarriage [5]. Furthermore, many drugs routinely used to treat migraine are not licensed for use in pregnancy and

Some are associated with increased risk of teratogenicity [6].

Some women may still require treatment during pregnancy to manage symptoms and prevent attacks and therapeutic options are often limited.

In this review, we examine the risks associated with migraine and treatment of migraine in pregnancy. It remains important to discuss these risks with patients and use them to guide management during pregnancy.

## 2. The Course of Migraine during Pregnancy

A review of observational studies found the majority of women (60–70%) report that migraine resolves during pregnancy, with rates of remission highest in the second and third trimesters [7]. This is thought to be due to a number of factors. For instance, oestrogen and endogenous opioids rise during pregnancy, raising the pain threshold. In addition, there are no longer menstruation-related hormonal fluctuations, which are a major trigger for migraine attacks [8]. Remission of migraine symptoms has been reported to be more common in those with migraine without aura than in those with aura [8].

Less often, migraines can occur for the first time during pregnancy [9]. A study on the course of migraine during pregnancy found that 10–14% of women with migraine experienced it for the first time in pregnancy [9]. Patients presenting with new onset of headache in pregnancy should be carefully evaluated [10].

Migraine symptoms commonly reappear early during the postpartum period. This is thought to be related to the rapid drop in oestrogen levels following delivery. In addition, poor sleep, stress and anxiety that can accompany the postpartum period are all common triggers of migraine [8].

## 3. Management of Headache in Pregnancy

Most headaches presenting in pregnancy are primary, with the majority being migraine or tension-type headaches. Reassuring features (green flags) of headaches presenting in pregnancy include: the presence of similar headache prior to pregnancy (such as migraine); presence of headache-free days; and headache occurring and resolving more than one week prior to presentation [10].

However, pregnancy is also a risk factor for some secondary headaches including: pre-eclampsia; sinus thrombosis; pituitary apoplexy; and posterior reversible encephalopathy. Concerning features (red flags) of headache that warrant urgent specialist referral include: sudden onset “thunderclap” headache; signs of raised intracranial pressure, such as papilloedema; focal neurological or visual deficits; systemic illness (for example, fever or meningism); or recent head or neck injury.

Initial examination and investigation should therefore include a neurological examination, fundoscopy, blood pressure and urine dip.

Once a secondary cause of headache has been excluded, then symptom control is based on what phenotypically the headache best represents [2]. In most women this will be migraine and tension-type headache.

## 4. Management of Migraine in Pregnancy

As with any patient with migraine, patients experiencing migraine in pregnancy should be advised of lifestyle changes that may improve symptoms. These include the avoidance of triggers, the management of stress, good sleep hygiene, staying hydrated, regular meals and exercise [11].

First line therapy for the acute management of migraine (Table 1) should be with paracetamol [12]. Non-steroidal anti-inflammatory drugs (NSAIDs) can be used in early pregnancy, although they are associated with a potential risk of miscarriage if taken around conception [13]. NSAIDs are also advised against in the third trimester, due to the increased risk of premature closure of ductus arteriosus [14,15,16]. Frequent use of opiates should be avoided due to impact on gastric motility, risk of medication overuse headache [17] and risk of neonatal abstinence syndrome in the newborn [18]. Triptans are currently considered safe in pregnancy (in particular sumatriptan) [12,17], but these should not be commenced for the first time during pregnancy.

It remains unclear whether newer acute migraine treatments, including Gepants and Ditans, are safe in pregnancy and until further data is available should be avoided [19].

If anti-emetics are required, cyclizine, prochlorperazine, ondansetron, domperidone and metoclopramide can be taken during pregnancy [10].

Preventative therapy (Table 1) should be considered if triptans are required more than twice a week. Propranolol and amitriptyline may be considered during pregnancy and should be started at the lowest possible dose and slowly increased according to benefits and tolerability. Although not licensed, botulinum toxin (Botox) for the treatment of chronic migraine [20] is considered safe in pregnancy and is often continued [21,22]. Greater occipital nerve blocks are particularly useful in pregnancy and have been shown to provide longer term relief from headache symptoms and reduce the need for other medications [17].

Newer drugs that target the calcitonin gene-related peptide (CGRP) system [23], including monoclonal antibody therapies and CGRP receptor antagonists (Gepants); should be avoided until safety data become available.

Vestibular migraine, which may occur in the absence of headache and is characterized by vestibular symptoms (e.g., vertigo, dizziness and giddiness) and often associated with other migraine symptoms (e.g., nausea, brain fog and fatigue), may be difficult to distinguish from the normal symptoms of early pregnancy [24]. Treatment of the vestibular component can also be challenging as certain effective drugs (e.g., Cinnarizine) are contraindicated in pregnancy. Some patients may benefit from vestibular rehabilitation.

Postural orthostatic tachycardic syndrome (POTS), is characterized by orthostatic intolerance (e.g., palpitations, tachycardia and (pre)syncope) and often occurs in association with migraine and is common in early pregnancy [25]. In the majority of cases, simple advice (e.g., increased fluid and salt intake) and non-pharmacological interventions (e.g., compression stockings) helps to control symptoms. Some women may also benefit from betablockers (e.g., Propranolol).

Alternative therapies to treat migraine such as acupuncture, mindfulness and meditation, biofeedback and cognitive behavioural therapy can be discussed in women whose symptoms do not improve [26]. Some women also benefit from neuromodulation, including supraorbital nerve stimulation, which is safe in pregnancy [27]. Regular moderate intensity physical activity should also be encouraged in pregnancy and if tolerated may help alleviate migraine in some patients [28].

Management of migraine in breastfeeding mothers provides significantly more treatments options and except for a few drugs (Table 1), where risks are already known or there is a paucity of safety data, most acute and preventatives therapies can be used.

## 5. Known Risks Related to Migraine in Pregnancy

There is evidence to suggest that migraine impacts pregnancy outcomes. In particular, a link between migraine and pre-eclampsia, a disorder of the placenta characterised by new-onset hypertension and end-organ damage, has been well described. An umbrella review with an updated systematic review found a twofold increase in odds of pre-eclampsia associated with migraine (pooled OR 2.05 (1.47–2.84)) [5].

The effects of migraine outside the brain may be due to endothelial dysfunction. Endothelial cells line blood vessel walls, forming a physical barrier and playing a role in clotting, vascular tone and inflammation. In people with migraine, endothelial dysfunction is thought to be mediated by oxidative stress. This leads to inflammation, impaired vascular reactivity and thrombosis [29]. Endothelial dysfunction has been suggested as one of the underlying causes of pre-eclampsia [30], suggesting this may be a potential mechanism behind the associations between migraine and this condition.

The umbrella review and updated systematic review also showed that women with migraine had a higher odds of preterm birth (pooled OR of 1.26 (1.21–1.32)). However, it should be noted that no distinction was made between spontaneous and medically indicated preterm birth, meaning the interpretation of this finding with regards to potential underlying mechanisms is difficult. Further research is needed to understand this [5].

A systematic review and meta-analysis examining the association between maternal chronic medical conditions and peripartum mental illness found that maternal migraine was associated with increased odds of peripartum mental illness (OR 1.75 (1.20–2.54)) [31]. Migraine is recognised to have an association with depressive disorders. This relationship is thought to be multifactorial and bidirectional. Hypothesised biological mechanisms include shared genetic variants, neurotransmitter dysfunction and pituitary axis dysfunction [32]. Specific to peripartum mental illness, psychosocial factors including managing migraine symptoms may increase the risk of postpartum depression. In addition, it is suggested that increased sensitivity to the postpartum decline in oestrogen may underly both migraine and postpartum depression [33].

There is some evidence that migraine may also be associated with an increased risk of miscarriage. A Danish cohort study reported a significant 10% increase in odds of miscarriage associated with migraine (adjusted prevalence ratio 1.10 (1.05–1.15)) [34]. Conversely, a Norwegian registry linkage study and a US prospective cohort study of pregnancy planners reported much smaller, non-significant associations between history of migraine and miscarriage. Similarly to pre-eclampsia, it is suggested that endothelial dysfunction may also be a common pathology between migraine and miscarriage [35]. There is also evidence of an association between some of the drugs used in the management of migraine and miscarriage (see below).

Ischemic stroke occurs in approximately 12 per 100,000 pregnancies [36] and although uncommon, the consequences to both mother and child can be devastating. In the United States, stroke accounted for approximately 8% of maternal deaths in pregnancy and postpartum [37] and remains a leading cause of major morbidity. Multiple studies have demonstrated that migraine is associated with up to a 15-fold increased risk of maternal stroke [38]. Suggested pathophysiological mechanisms include the increased risk of hypertensive disorders in pregnancy of women with migraine [39]. Given this association, correctable stroke risk factors, including smoking, obesity and hypertension, should be discussed during pregnancy planning.

## 6. Risks Associated with Migraine Medications in Pregnancy

A large UK pregnancy registry of approximately 1.4 million pregnancies recently showed that over the past two decades, the recorded rates of prescribing migraine medication in pregnancy has increased [5]. Potential harmful drug exposure has been reported to be higher in the first trimester, when women may not be aware they are pregnant [40]. Quantifying the potential risks of certain migraine medications in pregnancy remains important and guides management.

Pregnant women are advised to avoid many of the commonly prescribed migraine preventatives. Certain antiepileptics, including sodium valproate and topiramate, are contra-indicated due to an increased risk of congenital anomalies [41,42,43]. In the UK, the Medicines and Healthcare products Regulatory Agency (MHRA) has introduced new safety measures, including ‘pregnancy prevention programmes’ to avoid use of these drugs in pregnancy [44,45]. Candesartan is contra-indicated in the second and third trimesters due to an increased risk of severe fetopathy [46]. Although no specific risks associated with pizotifen use in pregnancy have been identified, available data are limited and its use in pregnancy is not recommended [47].

In a meta-analysis comparing triptan exposed women with healthy controls, Marchenko et al. found a threefold increase in the odds of miscarriage in women taking triptans (pooled OR 3.54 (2.24–5.59)) [48]. However, some of this association could be attributed to a possible association between migraine itself and miscarriage, as noted above. When comparing triptan exposed women with migraine controls, this study found a smaller, non-significant increase in the odds of miscarriage (1.27 (95% CI 0.58–2.79)). This was based on the results of two small studies which included 360 women, which may account for the wide confidence intervals. Results from a nested case-control in a published since this meta-analysis showed that, in comparison to healthy controls, women exposed to triptans during pregnancy had a higher odds of miscarriage (aOR: 1.63 (95% CI 1.34–1.98)) [49]. Current evidence is not suggestive of an association between triptan exposure and major congenital anomalies [48,50].

Caution has been advised with the use of CGRP inhibitors, a relatively new monoclonal antibody for the management of migraine, due to CGRP blockade having been found to cause an increase in systolic blood pressure and foetal mortality in pregnant animal models [51,52]. Although such risks have not been reported in humans exposed to CGRP inhibitors during pregnancy, further pharmacovigilance studies are required to confirm their safety [53,54].

## 7. Conclusions

Due to its high prevalence amongst women of reproductive age, it is important to understand how migraine and its associated treatments impact on pregnancy outcomes.

More detailed prospective studies, which carefully chart pregnancy outcomes in association with migraine severity and disease burden and associated medication use are therefore warranted and will help identify women who are at increased risk. The use of robust registry-based safety studies of migraine drugs in pregnancy are also warranted [55], especially for newer migraine drugs, including CGRP antagonists.

A recent survey of treatment practices of women’s healthcare providers highlighted the varying degrees of comfort when managing migraine in pregnancy and the need for further education [56]. More research is also needed to explore the ideas, concerns and expectations of women with migraine during pregnancy planning and pregnancy itself.

The pathophysiological mechanisms linking migraine to pregnancy related complications remain poorly understood. A notable example is the increased incidence of pregnancy related hypertensive disorders, including pre-eclampsia, which are associated with complications of pregnancy, including maternal stroke. In particular, a better understanding of the effects of migraine and medication used to treat migraine on placental physiology is required.

## Figures and Tables

**Table 1:** Safe acute headache and migraine preventative medication in pregnancy and breastfeeding.

Therapy	Pregnancy	Breastfeeding
**Acute medication**		
Paracetamol	✓	✓
NSAIDs	✓ stop by 30 weeks	✓
Opiates	✓ (use sparingly)	✓ (use sparingly)
Aspirin	✓ (low dose)	✓ (low dose)
Triptans (e.g Sumatriptan)	✓	✓
Gepants	x (no safety data)	x (no safety data)
Ditans	x (no safety data)	x (no safety data)
**Anti-emetics**		
Cyclizine	✓	x
Prochlorperazine,	✓	✓ (use sparingly)
Ondansetron,	✓	x
Domperidone	✓	✓
Metoclopramide.	✓	✓ (use sparingly)
**Migraine preventatives**		
Amitriptyline	✓	✓
Propranolol	✓	✓
Candesartan	x	✓ With caution in early new-borns
Topiramate	x	✓
Pizotifen	x	x
Onabotulinumtoxina (Botox)	✓	✓
Nerve blocks	✓	✓
Anti-CGRP antibody therapy	x (no safety data)	x (no safety data)
Gepants	x (no safety data)	x (no safety data)
**Neuromodulation**		
Cefaly supraorbital nerve stimulator	✓	✓
Gammacore external vagus nerve stimulator	X (no safety data)	✓

Key: NSAIDs, non-steroidal anti-inflammatory drugs; CGRP, calcitonin gene-related peptide.
